# Supplementary material for: Impaired mRNA splicing and proteostasis in preadipocytes in obesity-related metabolic disease
Source: eLife. 2021 Sep 21;10:e65996. doi: 10.7554/eLife.65996 (PMC8545398; doi:10.7554/eLife.65996)
Supplement: Supplementary file 1. — r = Spearman’s correlation coefficient. *P < 0.05, **P < 0.01, ***P < 0.001. Normality distribution was determined by Shapiro-Wilk normality test. [file elife-65996-supp1.docx]

**Supplementary file 1.**

|  | **SC Preadipocytes** | | **OM Preadipocytes** | |
| --- | --- | --- | --- | --- |
|  | **r** | ***P value*** | **r** | ***P value*** |
| **Age (years)** | -0.024 | 0.875 | 0.240 | 0.113 |
| **Weight (kg)** | -0.373 | 0.023 ***** | -0.461 | 0.004 ****** |
| **Height (m)** | -0.147 | 0.386 | -0.152 | 0.369 |
| **BMI (kg/m^2^)** | -0.518 | 0.001 ******* | -0.593 | <0.0001 ******* |
| **Fat mass (%)** | -0.585 | 0.024 ***** | -0.714 | 0.004 ****** |
| **Lean mass (%)** | -0.093 | 0.741 | 0.696 | 0.005 ****** |
| **Water mass (%)** | -0.161 | 0.567 | 0.436 | 0.105 |
| **Waist circumference (cm)** | -0.201 | 0.341 | -0.204 | 0.352 |
| **Systolic pressure (mm/Hg)** | -0.617 | 0.001 ****** | -0.127 | 0.553 |
| **Diastolic pressure (mm/Hg)** | -0.468 | 0.021 ***** | -0.053 | 0.805 |
| **Fasting glucose (mg/dL)** | -0.324 | 0.028 ***** | -0.406 | 0.005 ****** |
| **Fasting insulin (mU/L)** | -0.418 | 0.019 ***** | -0.427 | 0.017 ***** |
| **HbA1c (%)** | -0.417 | 0.007 ****** | -0.442 | 0.004 ** |
| **HOMA-IR (units)** | -0.546 | 0.002 ****** | -0.512 | 0.003 ** |
| **Total cholesterol (mg/dL)** | 0.112 | 0.474 | 0.075 | 0.632 |
| **LDL cholesterol (mg/dL)** | 0.160 | 0.365 | 0.074 | 0.679 |
| **HDL cholesterol (mg/dL)** | 0.030 | 0.865 | 0.116 | 0.514 |
| **Triglycerides (mg/dL)** | -0.170 | 0.281 | -0.337 | 0.029 ***** |
| **Free fatty acids (mmol/L)** | 0.239 | 0.262 | 0.431 | 0.036 ***** |
| **C-reactive protein (mg/L)** | -0.225 | 0.259 | -0.530 | 0.004 ****** |
| **Uric acid (mg/dL)** | 0.047 | 0.782 | 0.107 | 0.527 |
