# Supplementary material for: Impaired mRNA splicing and proteostasis in preadipocytes in obesity-related metabolic disease
Source: eLife. 2021 Sep 21;10:e65996. doi: 10.7554/eLife.65996 (PMC8545398; doi:10.7554/eLife.65996)
Supplement: Supplementary file 2. — NG, normoglycemic; T2D, type 2 diabetes; LDL, low-density lipoprotein; HDL, high-density lipoprotein; HOMA-IR, homeostasis model assessment of insulin resistance. aaP <0.01, aaaP <0.001 vs. Lean; bP <0.05 vs. NG Obese. One-way ANOVA with Tukey’s multiple comparisons test or Kruskal-Wallis with Dunn’s multiple comparisons test (for parametric or non-parametric data, respectively) were used. Normality distribution was determined by Shapiro-Wilk normality test. [file elife-65996-supp2.docx]

**Supplementary file 2.**

|  | **Lean** | **NG Obese** | **T2D Obese** |
| --- | --- | --- | --- |
|  |  |  |  |
| **N** | 18 | 15 | 10 |
| **Gender (female/male)** | 10 / 8 | 9 / 6 | 6 / 4 |
| **Antidiabetic therapy (n, %)** | 0 (0) | 0 (0) | 7 (70) |
| **Age (years)** | 51 ± 13 | 54 ± 14 | 60 ± 8 |
| **Weight (kg)** | 65.6 ± 8.7 | 92.2 ± 10.7 **^aaa^** | 90.0 ± 11.7 **^aaa^** |
| **Height (m)** | 1.68 ± 0.07 | 1.66 ± 0.10 | 1.68 ± 0.12 |
| **Body mass index (kg/m^2^)** | 23.3 ± 2.1 | 33.6 ± 2.7 **^aaa^** | 32.1 ± 2.9 **^aaa^** |
| **Systolic pressure (mm/Hg)** | 125.1 ± 17.3 | 138.7 ± 17.5 | 142.2 ± 15.0 |
| **Diastolic pressure (mm/Hg)** | 74.2 ± 8.3 | 76.2 ± 9.8 | 81.6 ± 12.3 |
| **Fasting glucose (mg/dL)** | 88.0 ± 13.2 | 97.3 ± 15.3 | 121.6 ± 41.4 **^aa, b^** |
| **Fasting glucose (mmol/L)** | 5.51 ± 0.96 | 5.84 ± 0.80 | 8.24 ± 5.61 **^aa, b^** |
| **Fasting insulin (mU/L)** | 11.4 ± 2.2 | 12.7 ± 6.7 | 18.4 ± 1.3 |
| **HOMA-IR (units)** | 2.82 ± 0.94 | 3.28 ± 1.81 | 6.91 ± 5.08 |
| **Total cholesterol (mg/dL)** | 184.7 ± 33.6 | 192.8 ± 51.8 | 172.2 ± 24.6 |
| **LDL cholesterol (mg/dL)** | 104.1 ± 37.1 | 124.8 ± 43.9 | 98.7 ± 10.5 |
| **HDL cholesterol (mg/dL)** | 54.0 ± 11.0 | 49.7 ± 16.1 | 43.0 ± 14.0 |
| **Triglycerides (mg/dL)** | 106.7 ± 62.1 | 142.3 ± 60.1 | 226.6 ± 162.5 **^aa^** |
| **Urid acid (mg/dL)** | 4.65 ± 0.68 | 5.13± 0.97 | 6.48 ± 0.96 **^aa, b^** |
|  |  |  |  |
