# Supplementary material for: Impaired mRNA splicing and proteostasis in preadipocytes in obesity-related metabolic disease
Source: eLife. 2021 Sep 21;10:e65996. doi: 10.7554/eLife.65996 (PMC8545398; doi:10.7554/eLife.65996)
Supplement: Supplementary file 3. — bp, base pairs. [file elife-65996-supp3.docx]

**Supplementary file 3.**

|  | **Primer sequences (5’ → 3’)** | |  |
| --- | --- | --- | --- |
| **Gene (NCBI reference)** | **Forward** | **Reverse** | **Size (bp)** |
|  |  |  |  |
| ***Hsa ACTB (NM_001101.3)*** | ACTCTTCCAGCCTTCCTTCTT | CAGTGATCTCCTTCTGCATCC | 176 |
| ***Hsa ADIPOQ (NM_009605.4)*** | TGGAGAAGCCGCTTATGTGT | GTCCCGGAATGTTGCAGTAG | 159 |
| ***Hsa BiP (X87949.1)*** | CTATGAAGCCCGTCCAGAAA | GTCGAGCCACCAACAAGAAC | 85 |
| ***Hsa BSCL2-1 (NM_001122955.3)*** | TCAGATCCTGGAGCATACCAA | CAGGCAGTGAACTAGCGACA | 94 |
| ***Hsa BSCL2-2/3 (KU178595.1)*** | GCCTCCTGCTATTTGGCTTT | TCTTCAGGGCTCTCACCATC | 309 |
| ***Hsa CCL2 (NM_002982.4)*** | CTGGACAAGCAAACCCAAAC | GGGGAAAGCTAGGGGAAAA | 90 |
| ***Hsa CIDEB-1 (NM_001318807.2)*** | GCCTACAGCCTGCCTCTTTT | TTGTGTTTGAGGTGGGGTCT | 72 |
| ***Hsa CIDEB-2 (NM_014430.3)*** | GGGCCTACAGGACACAGAAA | TGGACGTGACAGAGATGTGAA | 237 |
| ***Hsa CIDEC-5 (NM_001321142.2)*** | GAGGCTGTGAGGGGGAGAA | CTTGTCAGCTGGACTGCGTT | 154 |
| ***Hsa CIDE_total_ (NM_001321142.2)*** | ATGGGAGAGAGGAGGCTTTG | CACTGACACATGCCTGGAGA | 147 |
| ***Hsa DERL1 (NM_001134671.2)*** | AAGACAAGCAGCGGATGAAG | AGGAAACAAACACCCAGCAA | 175 |
| ***Hsa* *FABP4 (NM_001442.3)*** | TGCAGCTTCCTTCTCACCTT | GGCAAAGCCCACTCCTACTT | 134 |
| ***Hsa FABP5 (DN991192)*** | GAAGGAGCTAGGAGTGGGAATAG | TCTCTCCCAGGGTACAAGAAAA | 146 |
| ***Hsa GAPDH (NM_002046.5)*** | AATCCCATCACCATCTTCCA | AAATGAGCCCCAGCCTTC | 122 |
| ***Hsa HPRT (S79316.1)*** | TAATCCAGCAGGTCAGCAAAG | CTGAGGATTTGGAAAGGGTGT | 157 |
| ***Has HRD1 (AB085847.1)*** | TGATGGGCAAGGTGTTCTTT | GCCAGTGGAAACATTTGAGG | 177 |
| ***Hsa KI67 (NM_002417)*** | GACATCCGTATCCAGCTTCCT | GCCGTACAGGCTCATCAATAAC | 233 |
| ***Hsa γORF4* (Aprile et al., 2014)** | AAACCCAAAACAACTTCCCG | CTTGCAGTGGGGATGTCTCA | 279 |
| ***Hsa PDGFRA (NM_006206.6)*** | CCTTTTTGTGACGGTCTTGG | AAAGGCTACATCTGGGTCTGG | 148 |
| ***Hsa PPARG-1* (Aprile et al., 2014)** | CGAGGACACCGGAGAGGG | TGTGGTTTAGTGTTGGCTTCTT | 69 |
| ***Hsa PPARG-2 (NM_015869.4)*** | AGGAGTGGGAGTGGTCTTCC | TTTTAACGGATTGATCTTTTGC | 255 |
| ***Hsa PPARG-3 (NM_138711.3)*** | TGGGTGTGTAGTCGTGGTACTTT | CTCGGTTACTCCCCGTTTCT | 82 |
| ***Hsa PPARG-4 (NM_005037.6)*** | GCCGTGGCCGCAGAAA | CCACGGAGCTGATCCCAAAG | 76 |
| ***Hsa PPARG_total_* (Aprile et al., 2014)** | ATGGCCACCTCTTTGCTCT | GAGAAGGAGAAGCTGTTGGC | 272 |
| ***Hsa RAD23A (D21235.1)*** | TGGCCCAAATCTTTCCATC | TGAGCTGATGTTAGCCCTTCTC | 100 |
| ***Hsa RNF185 (NM_152267.3)*** | CAACAGGACCCCAGAGAGAA | CCACCATCTCCAAATCCAAA | 101 |
| ***Hsa SEC61A1 (NM_013336.3)*** | GAGTGGACCTGCCAATCAAG | AGCTGAGAGCATTTGGGAGA | 146 |
| ***Hsa SEL1L (AF052059.1)*** | CCTGCCACTTCCCTTTTCTT | CCATTTCTGCTTCCTGCATC | 180 |
| ***Hsa SREBF1-1 (NM_001005291.3)*** | GACCGACATCGAAGGTGAAG | GGTCAAATAGGCCAGGGAAG | 158 |
| ***Hsa SREBF1_total_ (NM_001005291.3)*** | AGCTCAAGGATCTGGTGGTG | TGTGTTGCAGAAAGCGAATG | 93 |
| ***Hsa STT3A (NM_001278503.1)*** | AGCTATGGCAAACCGAACAA | CCTCCAAAAATGACCAGCAC | 153 |
| ***Hsa STT3B (NM_178862.2)*** | CCCTCCTCCTTTTTCTTCCTC | ACGACTTGTGCTTGCTCTCC | 153 |
| ***Hsa TGFB2 (NM­_000660.6)*** | GCAACAATTCCTGGCGATAC | CTAAGGCGAAAGCCCTCAA | 136 |
| ***Hsa UBQLN1 (NM_013438.4)*** | CAACACACCAGGAATGCAGA | CTTCTCATGTAGGGGGCAGA | 90 |
| ***Hsa UPK3B (NM_030570.3)*** | TGCACTCAGGAGGGTTTAGG | ATGGTGGAGCAGGAGAGACA | 135 |
| ***Hsa XBP1s (NM_001079539.1)*** | TGGATGCCCTGGTTGCT | CACCTGCTGCGGACTCA | 87 |
| ***Hsa XBP1u (NM_005080.3)*** | CGCAGCACTCAGACTACGTG | CTGGGTCCAAGTTGTCCAGA | 147 |
| ***Mmu PRPF8 (NM_138659.2)*** | TACATGACGGCCAAGAACAA | TGCAATCCCAGTACAAGCAA | 146 |
|  |  |  |  |
